# Efficient Multiple Genome Modifications Induced by the crRNAs, tracrRNA and Cas9 Protein Complex in Zebrafish

**DOI:** 10.1371/journal.pone.0128319

**Published:** 2015-05-26

**Authors:** Hirohito Kotani, Kiyohito Taimatsu, Rie Ohga, Satoshi Ota, Atsuo Kawahara

**Affiliations:** Laboratory for Developmental Biology, Center for Medical Education and Sciences, Graduate School of Medical Science, University of Yamanashi, Shimokato 1110, Chuo, Yamanashi, Japan; Osaka University, JAPAN

## Abstract

The type II clustered regularly interspaced short palindromic repeats (CRISPR) associated with Cas9 endonuclease (CRISPR/Cas9) has become a powerful genetic tool for understanding the function of a gene of interest. In zebrafish, the injection of Cas9 mRNA and guide-RNA (gRNA), which are prepared using an *in vitro* transcription system, efficiently induce DNA double-strand breaks (DSBs) at the targeted genomic locus. Because gRNA was originally constructed by fusing two short RNAs CRISPR RNA (crRNA) and *trans*-activating crRNA (tracrRNA), we examined the effect of synthetic crRNAs and tracrRNA with Cas9 mRNA or Cas9 protein on the genome editing activity. We previously reported that the disruption of *tyrosinase* (*tyr*) by tyr-gRNA/Cas9 mRNA causes a retinal pigment defect, whereas the disruption of *spns2* by spns2-gRNA1/Cas9 mRNA leads to a cardiac progenitor migration defect in zebrafish. Here, we found that the injection of spns2-crRNA1, tyr-crRNA and tracrRNA with Cas9 mRNA or Cas9 protein simultaneously caused a migration defect in cardiac progenitors and a pigment defect in retinal epithelial cells. A time course analysis demonstrated that the injection of crRNAs and tracrRNA with Cas9 protein rapidly induced genome modifications compared with the injection of crRNAs and tracrRNA with Cas9 mRNA. We further show that the crRNA-tracrRNA-Cas9 protein complex is functional for the visualization of endogenous gene expression; therefore, this is a very powerful, ready-to-use system in zebrafish.

## Introduction

Recent remarkable innovations in genome editing technologies, such as transcription activator-like effector nucleases (TALENs) and the clustered regularly interspaced short palindromic repeats (CRISPR)/CRISPR-associated protein (Cas) system, enable us to induce genome modifications at targeted genomic loci [1,2,3,4,5,6,7]. These systems allow us to generate loss-of-function alleles by frameshift-mediated mutations and establish knock-in alleles using donor DNA in various model organisms, including zebrafish [8,9,10,11,12]. The CRISPR-mediated gene regulation system is established by using Cas9 transcriptional activator or repressor [13,14]. More recently, the engineered DNA-binding molecule-mediated chromatin immunoprecipitation (enChIP) using CRISPR system is developed to isolate specific genomic regions retaining molecular interactions [15,16].

The type II CRISPR system in *Streptococcus pyogenes* uses the Cas9 endonuclease and the two small RNAs target-recognizing CRISPR RNA (crRNA) and auxiliary *trans*-activating crRNA (tracrRNA), which are essential for the RNA-guided cleavage of invading foreign DNA [17]. In fact, crRNA recognizes the target DNA that is 20 bp in length followed by the protospacer adjacent motif (PAM) sequence NGG (N: any nucleotide). Recently, a guide RNA (gRNA), which contains complementary sequences to the target and a Cas9 interaction interface, was developed by fusing crRNA and tracrRNA [5,6]. In zebrafish, we and other groups have demonstrated that the injection of Cas9 mRNA and multiple gRNAs for the targeted genomic loci simultaneously induces DNA double-strand breaks (DSBs) at the target sites at a high frequency, leading to a frameshift-mediated targeted gene disruption [18,19]. Furthermore, two groups have reported the efficient CRISPR/Cas9-mediated knock-in of a donor DNA by the homology-independent DSBs repair system [20,21].

We usually prepared gRNA (102 nt) using an *in vitro* transcription system from gRNA expression vector. Because both crRNA (42 nt) and tracrRNA (69 nt) are very short RNAs, we prepared synthetic crRNAs and tracrRNA and investigated multiple genome modifications by the injection of multiple crRNAs and common tracrRNA with recombinant Cas9 protein. Furthermore, as an application of this system, we tried to visualize the expression of an uncharacterized gene using the crRNA-tracrRNA-Cas9 protein complex. In this study, we demonstrated that the crRNA-tracrRNA-Cas9 protein complex is considerably functional for inducing multiple genome modifications and for visualizing the expression of an endogenous gene in zebrafish, thereby demonstrating its potential as a simple, customizable and ready-to-use genome editing tool.

## Materials and Methods

### Ethics Statement

This study was conducted in accordance with the recommendations in the Fundamental Guidelines for Proper Conduct of Animal Experiment and Related Activities of the Ministry of Education, Culture, Sports, Science and Technology in Japan. The Institutional Animal Care and Use Committee of Yamanashi University approved this study (Approval Identification Number: A25-28). As the experimental protocol of anaesthesia and euthanasia, zebrafish were treated with 0.2 mg/ml ethyl 3-aminobenzoate methanesulfonate salt followed by rapid freezing.

### Guide RNA vector, synthetic crRNA, Cas9 protein and Cas9 expression plasmid

The annealed oligonucleotides for *spns2*-target1, *spns2*-target2 and *tyr* listed in the S1 Table were cloned into the *Bsm*BI site of the pCS2P-gRNA vector. The region of gRNA containing the T7 promoter sequence was amplified by PCR using the target amplification primers (gRNA-F: 5’-GAATTCTAATACGACTCAC-3’ and gRNA-R: 5’-AAAAGCACCGACTCGG-3’), and the resultant PCR product was purified from agarose gel using the MinElute Gel Extraction Kit (Qiagen). Individual spns2-gRNA1, spns2-gRNA2 and tyr-gRNA were transcribed from the purified PCR products using the MAXIscript T7 Kit (Life Technologies) followed by a phenol/chloroform extraction and ethanol precipitation. The annealed oligonucleotides for *s1pr2*-target1 and *s1pr2*-target2 listed in the S1 Table were cloned into the *Bsa*I site of the pDR274 vector. Plasmid DNA containing *s1pr2*-target1 or *s1pr2*-target2 was linearized by *Dra*I. s1pr2-gRNA1 and s1pr2-gRNA2 were transcribed from the linearized templates using the MAXIscript T7 kit followed by a phenol/chloroform extraction and ethanol precipitation. The sequences of the genomic target sites and oligonucleotides are listed in the S1 Table. The pCS2-hSpCas9 plasmid was kindly provided by Dr. Kinoshita (Kyoto University) [22]. To prepare the Cas9 mRNA, pCS2-hSpCas9 was linearized by *Not*I. The Cas9 mRNA was transcribed using the mMESSAGE mMACHINE SP6 kit (Life Technologies) and purified using the RNeasy Mini Kit (Qiagen). The individual synthetic crRNAs and synthetic tracrRNA listed in the S1 Table were obtained from FASMAC (S1 Table), and the recombinant Cas9 protein was obtained from Toolgen [23]. The Cas9 protein, crRNAs and tracrRNA were dissolved in sterilized water at concentrations of 2000 ng/μl, 25 ng/μl and 100 ng/μl, respectively, and stored at -80°C.

### Microinjection

We used a wild-type AB line and the stable transgenic line Tg(*cmlc2*:*eGFP*), which expresses an enhanced green fluorescent protein (eGFP) under the cardiac-specific promoter *cmlc2* [24]. Multiple crRNAs (25 pg each) and tracr-RNA (100 pg) with Cas9 protein (400 pg) or Cas9 mRNA (250 pg) were co-injected into 1–2 cell stage zebrafish embryos derived from AB or Tg(*cmlc2*:*eGFP*) fish. Multiple gRNAs (25 pg each) with Cas9 protein (400 pg) or Cas9 mRNA (250 pg) were co-injected into 1–2 cell stage zebrafish embryos derived from AB or Tg(*cmlc2*:*eGFP*) fish. To visualize the expression of the *ependymain related 1* (*epdr1*) gene, epdr1-crRNA (25 pg), Mbait-crRNA (25 pg), tracrRNA (100 pg) and Mbait-hs-eGFP (25 pg) with Cas9 protein (400 pg) were co-injected into 1–2 cell stage zebrafish embryos derived from AB.

### Preparation of genomic DNA, heteroduplex mobility assay (HMA) and sequencing analysis

To prepare the genomic DNA, uninjected, crRNAs/Cas9-injected, or gRNAs/Cas9-injected embryos (1 dpf or 2 dpf) were incubated in 108 μl of 50 mM NaOH at 98°C for 10 min. Then, 12 μl of 1 M Tris-HCl (pH 8.0) was added to the resultant solution. Genomic fragments at the target sites were amplified by PCR with TaKaRa Ex Taq (TaKaRa) and the locus-specific primers listed in the S2 Table. The PCR conditions were as follows: 35 or 40 cycles of 98°C for 10 sec, 55°C for 30 sec and 72°C for 30 sec. The resultant PCR amplicons were electrophoresed on a 15% polyacrylamide gel (Wako) [10]. To evaluate the mutation rates of the individual target sites, we sub-cloned the PCR products into the pGEM-T Easy vector (Promega). The plasmid DNAs containing the genomic fragments were prepared from individual colonies, and then, random sequencing was performed.

### Western blotting

Uninjected, crRNA-tracrRNA-Cas9 protein-injected or crRNA-tracrRNA-Cas9 mRNA-injected sixty embryos were incubated in 150 μl of the lysis buffer (20 mM Tris-HCl pH7.5, 140 mM NaCl, 1% Triton X-100, 1 μM E-64, 1 μM leupeptin, 1 μM aprotinin, 500 μM disodium dihydrogen ethylenediaminetetraacetate dehydrate, 500 μM 4-[2-aminoethyl]-benzenesulfonyl fluoride hydrochloride). The lysate (5 μl) was mixed with 1 μl of 6 X sample buffer solution (Nacalai tesque). Western blotting was performed as previously described [25]. Anti-Cas9 antibody (Clontech) and anti-γ-Tubulin antibody (GeneTex) were used. Proteins recognized by the primary antibody were visualized using the enhanced chemiluminescence system (GE Healthcare) after reacting with the horseradish peroxidase-conjugated anti-rabbit IgG (Thermo Scientific).

### Whole-mount *in situ* hybridization with anti-sense probe

The *epdr1* gene was isolated from the cDNA of 20 somite stage zebrafish using the following oligonucleotide primers: epdr1-F, 5’-GGAATTCAACATGTTGGTGTTTGTTGTTTTATGG-3’; and epdr1-R, 5’-GCTCTAGATCAGCAGTCAGATGTCATCCT-3’ (S2 Table). The resultant PCR products were digested by *EcoR*I and *Xba*I, and were inserted into an *EcoR*I-*Xba*I-cleaved pCS2P+ vector. Whole-mount *in situ* hybridization was performed using the combination of well-established digoxigenin-labeled antisense *epdr1* RNA and an α-digoxigenin alkaline phosphatase-conjugated antibody [26].

## Results

### Multiple efficient genome modifications by the crRNAs, tracrRNA and Cas9 protein complex

**Fig 1:**
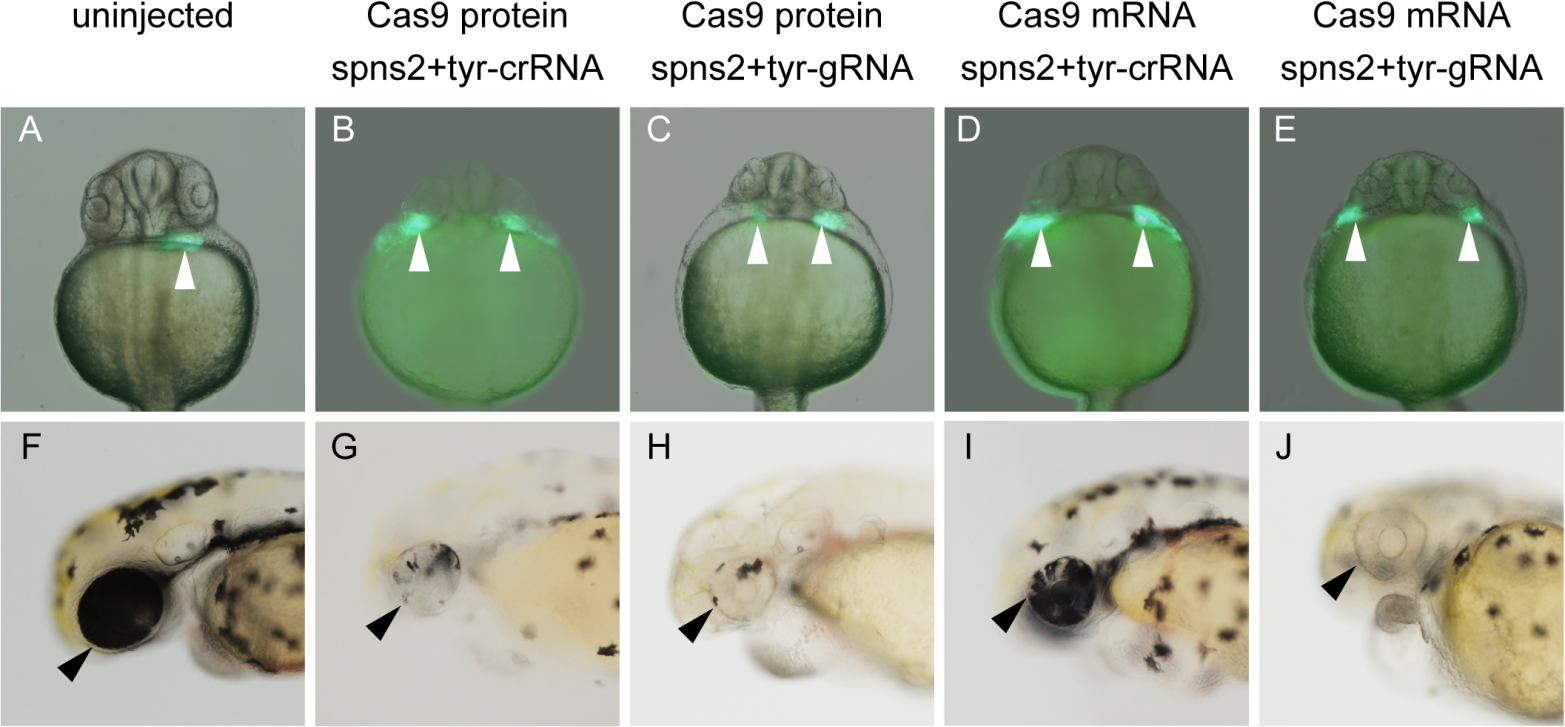
Phenotypic analysis in the embryos injected with two crRNAs, tracrRNA and Cas9 protein. (A, F) An uninjected embryo derived from Tg(*cmlc2*:*eGFP*) expressing eGFP in the cardiac cells. (B, G) Tg(*cmlc2*:*eGFP*)-derived embryos injected with spns2-crRNA1 (25 pg), tyr-crRNA (25 pg), tracrRNA (100 pg) and Cas9 protein (400 pg). (C, H) Tg(*cmlc2*:*eGFP*)-derived embryos injected with spns2-gRNA1 (25 pg), tyr-gRNA (25 pg) and Cas9 protein (400 pg). (D, I) Tg(*cmlc2*:*eGFP*)-derived embryos injected with spns2-crRNA1 (25 pg), tyr-crRNA (25 pg), tracrRNA (100 pg) and Cas9 mRNA (250 pg). (E, J) Tg(*cmlc2*:*eGFP*)-derived embryos injected with spns2-gRNA1 (25 pg), tyr-gRNA (25 pg) and Cas9 mRNA (250 pg). (A-E) The injection of the two crRNAs and tracrRNA with Cas9 protein or Cas9 mRNA (B and C) as well as the injection of two gRNAs with Cas9 protein or Cas9 mRNA (D and E) caused the cardiac progenitor migration defects at 1 day post-fertilization (dpf), whereas an uninjected embryo had a normal heart (A). White arrowheads indicate the position of the developing heart. (F–J) The injection of the two crRNAs and tracrRNA with Cas9 protein or Cas9 mRNA (G and H) as well as the injection of two gRNAs with Cas9 protein or Cas9 mRNA (I and J) caused pigmentation defects in the retinal epithelium at 2 dpf, whereas an uninjected embryo had retinal epithelial cells with normal pigmentation (F). Black arrowheads indicate the position of the eye. The embryos in (A), (B), (C), (D) and (E) correspond to the embryos in (F), (G), (H), (I) and (J), respectively. (A-E) Ventral view with anterior at the top. (F-J) Lateral view with anterior to the left and dorsal at the top.

Recently, we established an efficient targeted gene disruption system in zebrafish using gRNAs and Cas9 mRNA [19,27]. In this process, we constructed gRNA expression vectors for individual target genes and prepared gRNAs and Cas9 mRNA using an *in vitro* transcription system. Because the gRNA was originally constructed by fusing the two short RNAs crRNA and tracrRNA [5,6], we examined the effects of multiple synthetic crRNAs and tracrRNA on targeted genome modifications in zebrafish. At the same time, we compared the genome editing activity between the Cas9 mRNA and the recombinant Cas9 protein [23]. We chose the two genes, *tyrosinase* (*tyr*) and *spns2*, because the disruption of these genes results in obvious embryonic abnormalities. The disruption of *tyr* results in pigmentation defects in the eye [28]. The disruption of *spns2*, which is a transporter of the lipid mediator sphingosine-1-phosphate (S1P), causes cardiac progenitor migration defects, resulting in the two-hearts phenotype known as cardia bifida [24,29]. Moreover, we already possessed previously evaluated gRNAs (tyr-gRNA and spns2-gRNA1) with high genome editing activities for these two loci [19]. The two crRNAs spns2-crRNA1 and tyr-crRNA contain target sequences identical to those in spns2-gRNA1 and tyr-gRNA, respectively. We injected spns2-crRNA1 (25 pg), tyr-crRNA (25 pg) and tracrRNA (100 pg) with Cas9 protein (400 pg) or Cas9 mRNA (250 pg) into zebrafish embryos and found that the injections of two crRNA and tracrRNA with Cas9 protein or Cas9 mRNA simultaneously induced two distinct phenotypes, cardia bifida and an eye pigment defect, with high frequencies (Fig 1, S1 Fig and Table 1). These defects were identical to the phenotypes of the embryos injected with spns2-gRNA1 (25 pg) and tyr-gRNA (25 pg) with Cas9 protein (400 pg) or Cas9 mRNA (250 pg). When the Cas9 protein was injected into zebrafish without crRNAs-tracrRNA, we observed neither cardia bifida nor the eye pigment defect (S2 Fig and Table 1). Furthermore, embryos injected with crRNAs-trcrRNA without Cas9 protein exhibited normal heart and eye development (S2 Fig and Table 1). These results indicate that the complex consisting of crRNA, tracrRNA and Cas9 protein is essential for functioning in zebrafish.

**Table 1:** Summary of phenotypic analysis.

injection group	cardia bifida / embryos (%)	pigment defect / embryos (%)	number of total embryos
uninjected	0%	0%	n = 82
tracrRNA, spns2-crRNA1, tyr-crRNA, Cas9 protein	94 ± 3.3%	80 ± 1.7%	n = 64
spns2-gRNA1, tyr-gRNA, Cas9 protein	83 ± 5.9%	90 ± 7.1%	n = 80
tracrRNA, spns2-crRNA1, tyr-crRNA, Cas9 mRNA	76 ± 4.4%	58 ± 8.7%	n = 44
spns2-gRNA1, tyr-gRNA, Cas9 mRNA	72 ± 7.5%	83 ± 9.1%	n = 69
Cas9 protein	0%	0%	n = 68
Cas9 mRNA	0%	0%	n = 51
tracrRNA, spns2-crRNA1, tyr-crRNA	0%	0%	n = 48

Results of phenotypic analysis were indicated as the average of three independent experiments.

**Fig 2:**
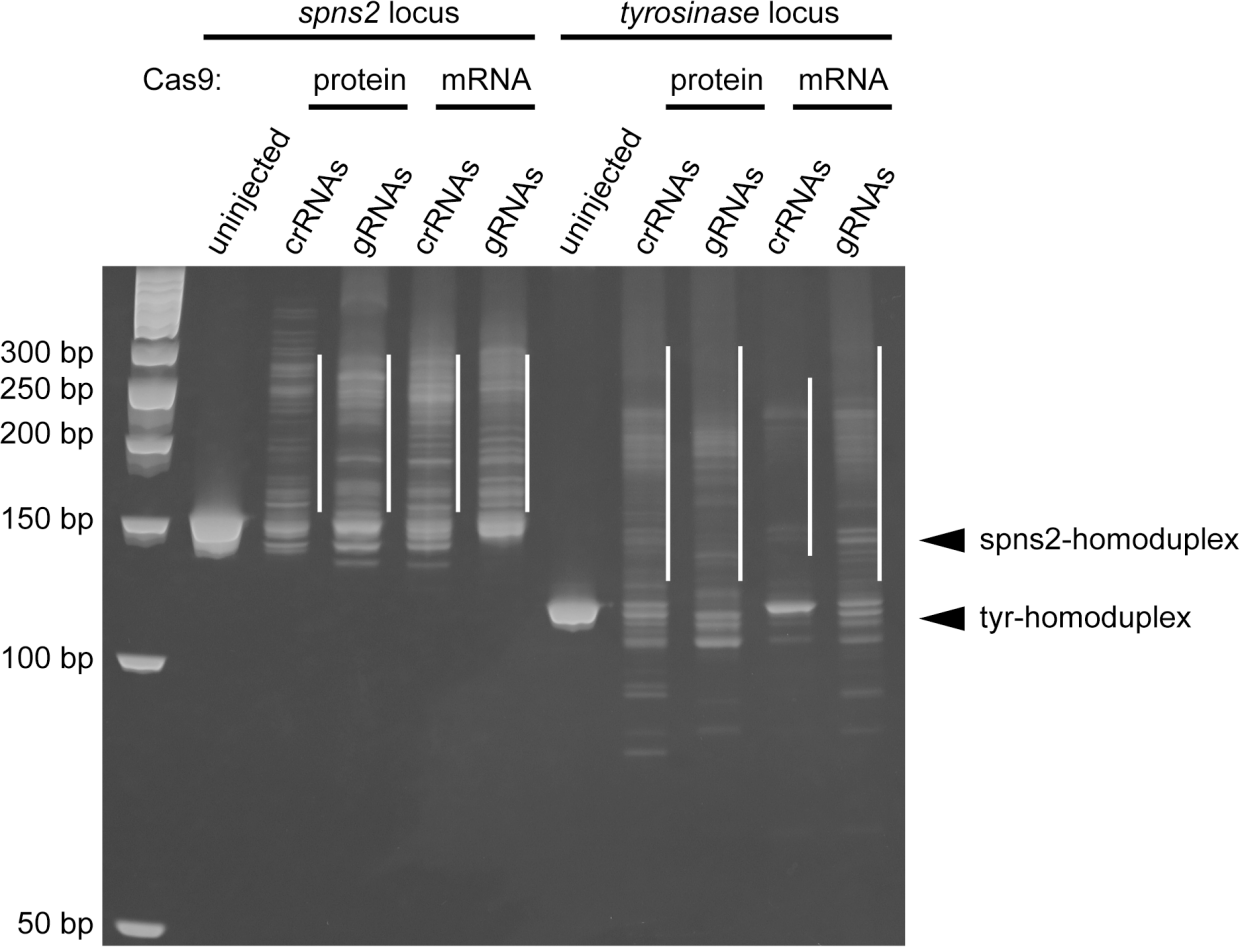
Heteroduplex mobility assay in the embryos injected with two crRNAs, tracrRNA and Cas9 protein. The *spns2*- and *tyr*-targeted genomic regions were amplified from the genomic DNA of individual embryos (as shown in Fig 1) by PCR using locus-specific primers. To perform the HMA, the resultant PCR amplicons were separated on a 15% polyacrylamide gel. The positions of the expected homoduplexes are indicated by the arrowheads and the multiple heteroduplexes are indicated by the white lines. The sizes of the DNA ladder markers are indicated by the base pairs at the left.

**Fig 3:**
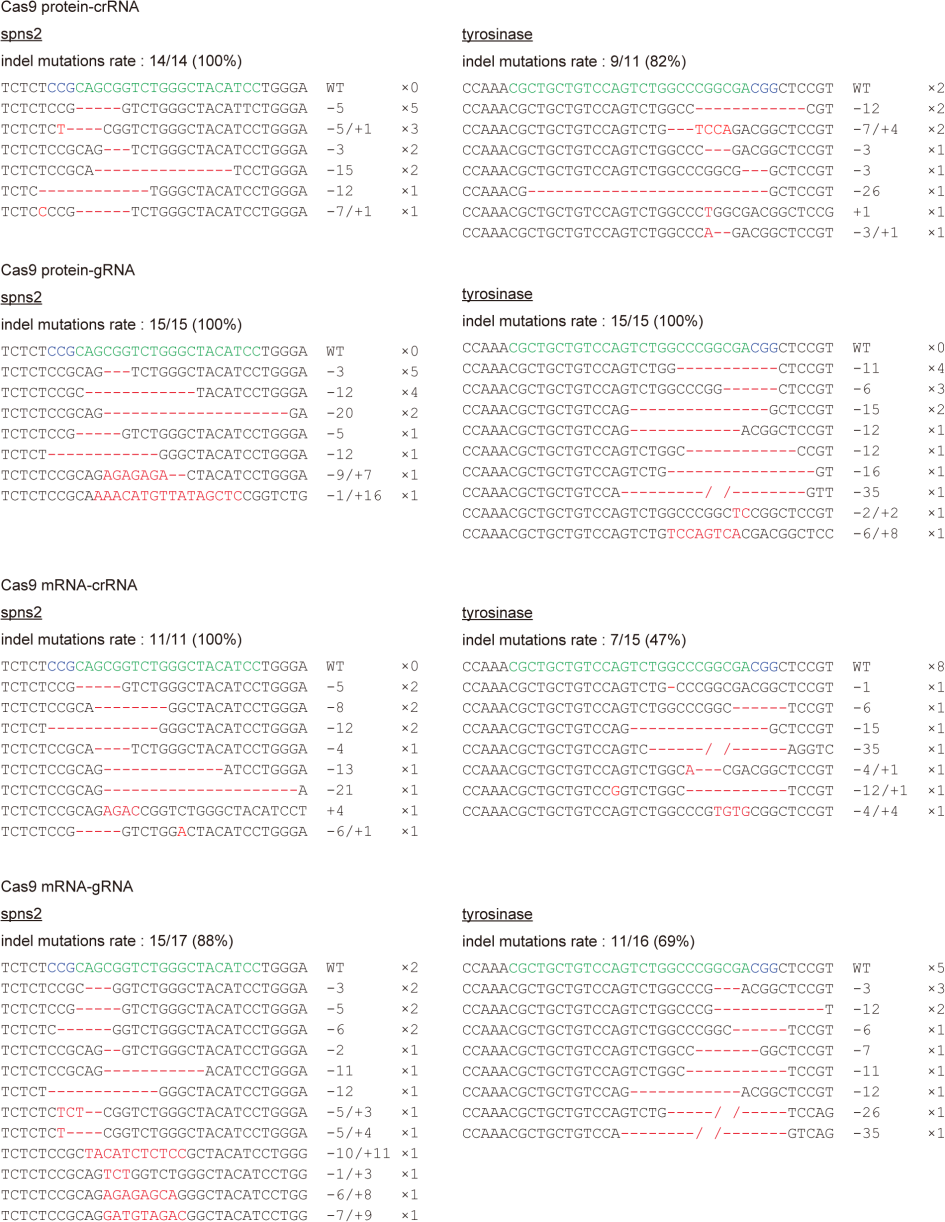
Sequence analysis of the genome modifications induced by two crRNAs, tracrRNA and Cas9 protein. PCR amplicons for the *spns2*- and *tyr*-target sites from the individual genomic DNA (as shown in Fig 2) were inserted into the pGEM-T Easy vector, and the inserted fragments derived from the individual PCR amplicons were randomly sequenced. The targeted genomic sequences and PAM sequences are indicated by the green and blue letters, respectively. The deleted and inserted nucleotides compared with the wild-type sequence (top row) are indicated by the red dashes and red letters, respectively. The numbers of nucleotides deleted (-) and inserted (+) are indicated to the right with the detection number. Slashes mean a gap in the genome sequence containing a large insertion or a large deletion.

We prepared the genomic DNA from the embryos that underwent the phenotypic analyses (Fig 1 and S1 Fig), and examined the genome editing activity using a heteroduplex mobility assay (HMA) as we previously described [10]. Indeed, the degree of heteroduplex formation was correlated with the genome editing activity. We found that the injection of two crRNAs and tracrRNA with the Cas9 protein or the Cas9 mRNA resulted in multiple heteroduplexes (white bars) above the homoduplexes (arrowheads) (Fig 2). The degree of heteroduplex formation is comparable to the degree of formation in the embryos injected with gRNAs and the Cas9 protein or the Cas9 mRNA. The sequencing analysis revealed that the tracrRNA-crRNAs-Cas9 (protein or mRNA) injections and the gRNAs-Cas9 (protein or mRNA) injections induced various indel mutations at a high frequency (Fig 3, S3 Fig and S4 Fig), indicating that the crRNAs-tracrRNA-Cas9 protein complex is a simple, ready-to-use genetic tool.

### Time course analysis of the genome modifications induced by Cas9 protein and Cas9 mRNA

**Fig 4:**
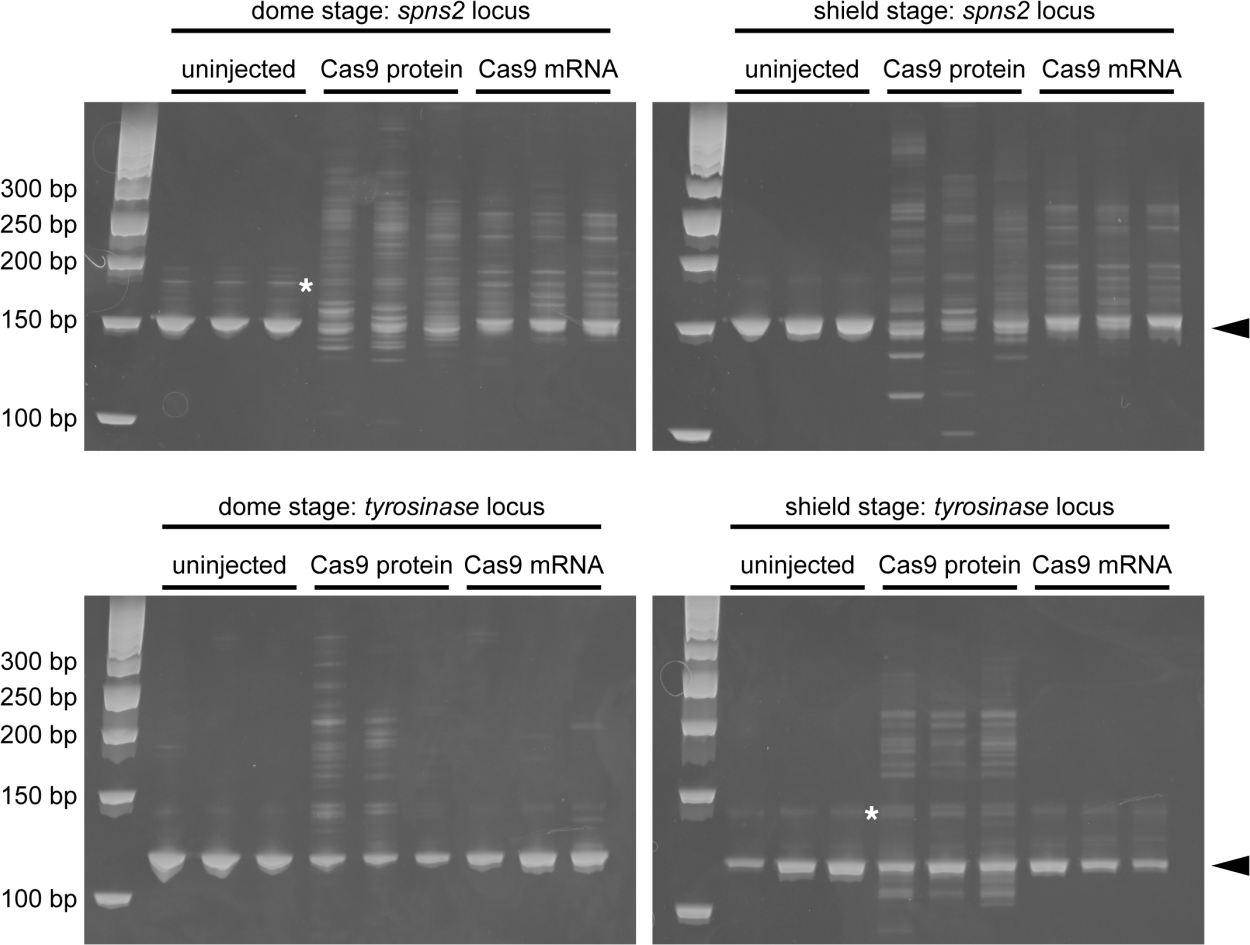
Time course analysis of the genome modifications induced by the Cas9 protein and Cas9 mRNA. Two crRNAs (spns2-crRNA1; 25 pg + tyr-crRNA; 25 pg) and tracrRNA (100 pg) were co-injected with Cas9 protein (400 pg) or Cas9 mRNA (250 pg) into zebrafish embryos and the genomic DNA was prepared from the dome stage embryos (4.3 hpf) or the shield stage embryos (6 hpf). Genome modifications induced by the Cas9 protein or Cas9 mRNA were assessed by a HMA. The positions of the expected homoduplexes are indicated by the arrowheads. Non-specific bands are indicated by the asterisks. The sizes of the DNA ladder markers are indicated by the base pairs at the left.

We predicted that the Cas9 protein rapidly functions compared to the Cas9 mRNA, because the Cas9 mRNA must be translated to function. We performed a time-course analysis of the genome modifications in embryos injected with crRNAs-tracrRNA-Cas9 protein or crRNAs-tracrRNA-Cas9 mRNA. Heteroduplexes in the embryos injected with the Cas9 protein were weakly detected in the *tyr* locus at the dome stage (4.3 hpf) and increased at the shield stage (6 hpf) (Fig 4), whereas the heteroduplexes in the embryos injected with the Cas9 mRNA were marginal. Furthermore, the formation of heteroduplexes in the embryos injected with the Cas9 protein was detected in the *spns2* locus at the dome and shield stage, which were more weakly detected in the embryos injected with the Cas9 mRNA. The sequencing analysis revealed that the genome modifications induced by the crRNAs-tracrRNA-Cas9 protein complex are more abundant than those induced by the crRNAs-tracrRNA-Cas9 mRNA complex (S5 Fig). Thus, consistent with the previous report on gRNA/Cas9 protein vs gRNA/Cas9 mRNA [23], the crRNAs-tracrRNA-Cas9 protein complex also induced genome modifications faster than those induced by the crRNAs-tracrRNA-Cas9 mRNA complex.

**Fig 5:**
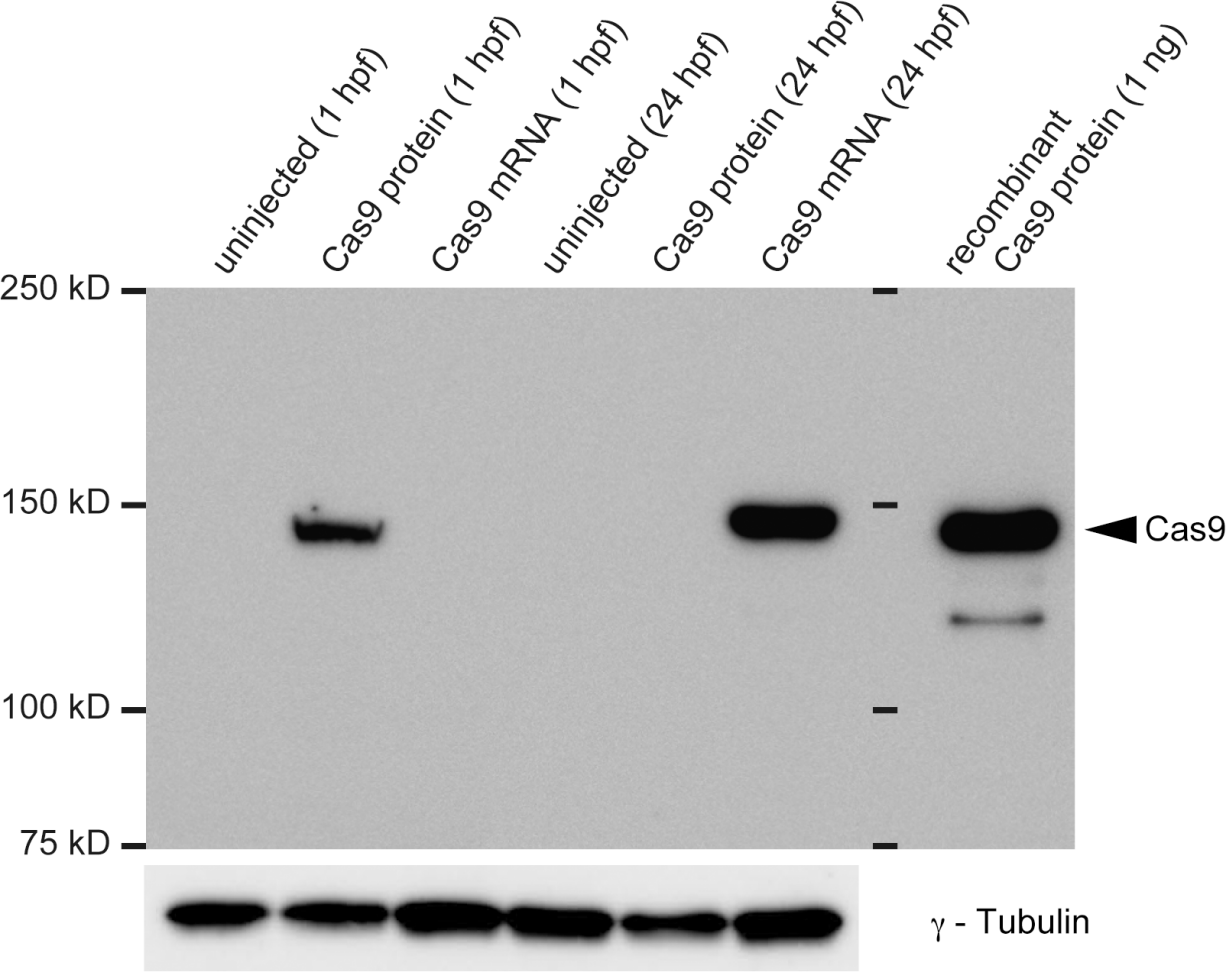
Detection of Cas9 protein by western blotting using anti-Cas9 antibody. Two crRNAs (spns2-crRNA1; 25 pg + tyr-crRNA; 25 pg) and tracrRNA (100 pg) were co-injected with Cas9 protein (400 pg) or Cas9 mRNA (250 pg) into zebrafish embryos and total whole body lysate was prepared from the embryos (1 hpf or 24 hpf). Western blotting was performed using anti-Cas9 protein and anti-γ-Tubulin antibodies. Recombinant Cas9 protein (1ng) was applied at the right as a control. Essentially similar results were obtained by three independent experiments. The positions of the indicated proteins are indicated by the arrowheads. The sizes of the protein markers are indicated by kD at the left.

To examine the Cas9 protein expression level, we performed western blotting using anti-Cas9 antibody. Cell lysates were prepared from uninjected, crRNAs-tracrRNA-Cas9 protein-injected or crRNAs-tracrRNA-Cas9 mRNA-injected embryos. We found that Cas9 protein was detected at 1 hpf and disappeared at 24 hpf in the lysate derived from the crRNAs-tracrRNA-Cas9 protein-injected embryos, whereas Cas9 protein was marginal at 1 hpf and significantly accumulated at 24 hpf in the lysate derived from the crRNAs-tracrRNA-Cas9 mRNA-injected embryos (Fig 5). We confirmed that the protein expression of γ-Tubulin was comparable among them.

### Multiple genome modifications induced by the crRNAs-tracrRNA-Cas9 protein were heritable

**Fig 6:**
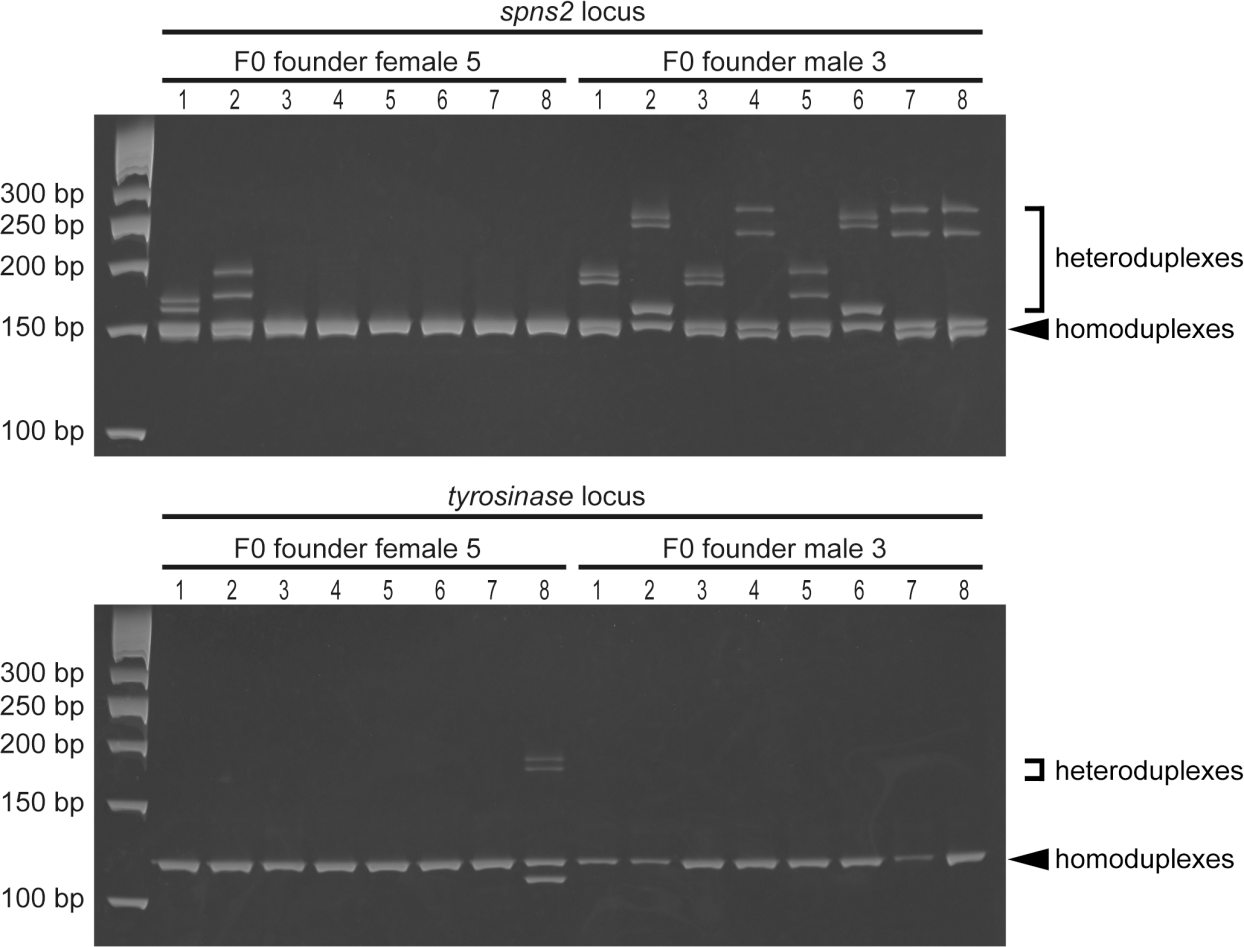
Detection of heteroduplexes at *spns2* and *tyr* loci by HMA in F1 embryos. F0 founder male 3 and female 5 were mated with wild-type fish and genomic DNA from individual F1 embryos was isolated at 1 dpf. PCR amplicons for *spns2* or *tyr* were electrophoresed on a 15% polyacrylamide gel. We observed heteroduplex bands containing mutant alleles (brackets) at *spns2* and *tyr* loci. The positions of the expected homoduplexes are indicated by the arrowheads. The sizes of the DNA ladder markers are indicated by the base pairs at the left.

We previously reported that the multiple genome modifications induced by spns2-gRNA1, tyr-gRNA with Cas9 mRNA are transmitted to next generation [19]. We injected spns2-crRNA1 (5 pg), tyr-crRNA (5 pg) and tracrRNA (20 pg) with Cas9 protein (80 pg) into zebrafish embryos to avoid embryonic lethality and obtained F0 founders. After the F0 founders were mated with wild-type fish, genomic DNA was prepared from individual embryos. We found that the heteroduplexes containing mutant alleles at both *spns2* and *tyr* loci were detected in F0 founder female 5, while the heteroduplexes containing mutant alleles at *spns2* locus, but not *tyr* locus, were observed in F0 founder male 3 (Fig 6). Thus, these results indicate that the multiple genome modifications induced by the crRNA-tracrRNA-Cas9 protein were heritable.

### Comparison of crRNAs and gRNAs on the Cas9 protein-induced genome modifications

**Fig 7:**
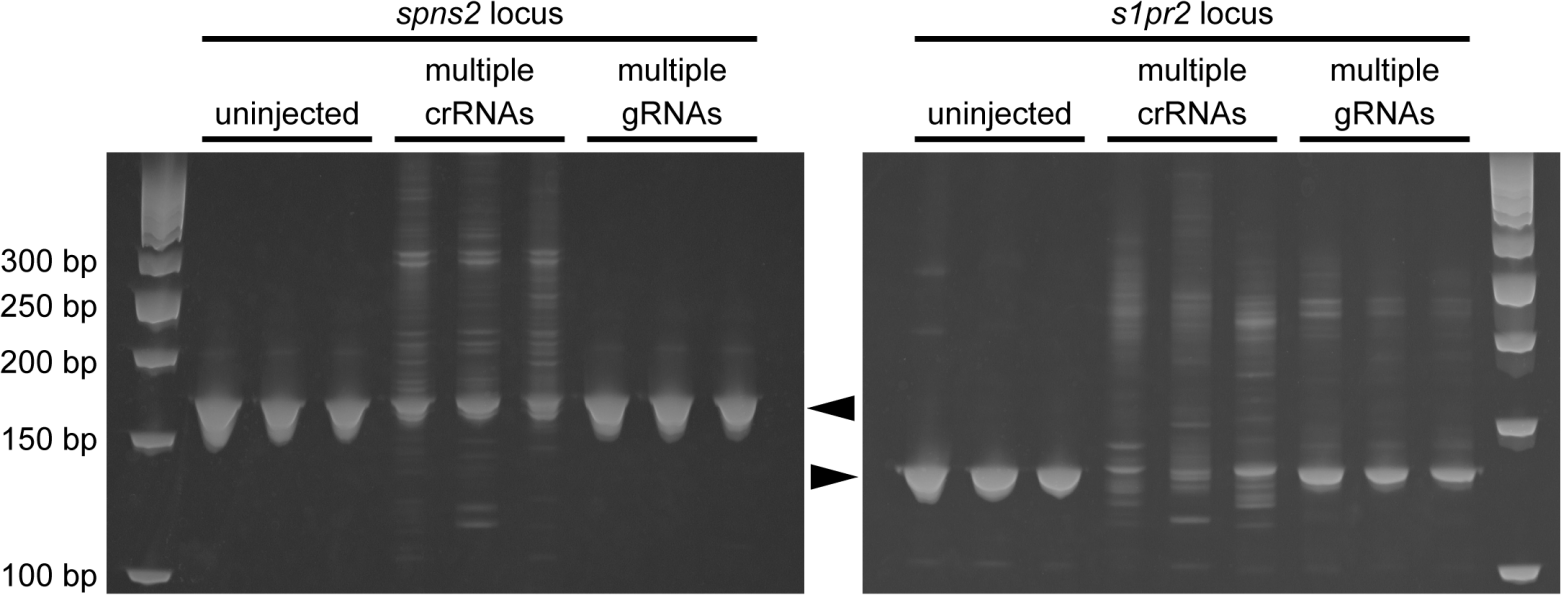
Comparison of the crRNAs and gRNAs on the Cas9 protein-mediated genome editing activity. Three crRNAs (spns2-crRNA2; 25 pg + s1pr2-crRNA1; 25 pg + s1pr2-crRNA2; 25 pg) plus tracrRNA (100 pg) or three gRNAs (spns2-gRNA2; 25 pg + S1PR2-gRNA1; 25 pg + S1PR2-gRNA2; 25 pg) were co-injected with Cas9 protein (400 pg) into zebrafish embryos and the genomic DNA was prepared from 24 hpf embryos. The genome modifications induced by the Cas9 protein were assessed by a HMA. Target sites for S1PR2-gRNA1 and S1PR2-gRNA2 are located within the binding sites of S1PR2-specific primers. The positions of the expected homoduplexes are indicated by the asterisks. The sizes of the DNA ladder markers are indicated by the base pairs at the left.

Because we already had several gRNAs (spns2-gRNA2 for the *spns2* coding region, S1PR2-gRNA1 and S1PR2-gRNA2 for the S1PR2 5’ non-coding region) with low genome editing activity, we prepared synthetic spns2-crRNA2, s1pr2-crRNA1, s1pr2-crRNA2 containing the respective identical target sequences and compared the genome editing activity between the crRNAs-tracrRNA and gRNAs. The disruption of *s1pr2* gene exhibits cardia bifida in zebrafish [30]. The Cas9 protein was co-injected with multiple crRNAs (spns2-crRNA2, s1pr2-crRNA1 and s1pr2-crRNA2) plus tracrRNA or multiple gRNAs (spns2-gRNA2, s1pr2-gRNA1 and s1pr2-gRNA2) into zebrafish embryos, and the genome editing activities were assessed by a HMA. In the *spns2* locus, multiple heteroduplexes in the embryos injected multiple crRNAs and tracrRNA with the Cas9 protein, were noticeably observed above the *spns2* wild-type homoduplexes compared with those in the embryos injected with multiple gRNAs and the Cas9 protein (Fig 7). Furthermore, the genome editing activity in the *s1pr2* locus, as determined by the formation of multiple heteroduplexes, was higher in the embryos injected with the crRNAs compared with those injected with gRNAs. We observed cardia bifida in the embryo injected with the crRNAs-tracrRNA-Cas9 protein injection, but not with the gRNAs-Cas9 protein injection (S6 Fig). These results suggest that synthetic crRNAs, instead of gRNAs, with tracrRNA is an alternative tool for efficient genome editing in zebrafish.

### Visualization of endogenous gene expression by the crRNAs, tracrRNA and Cas9 protein complex

**Fig 8:**
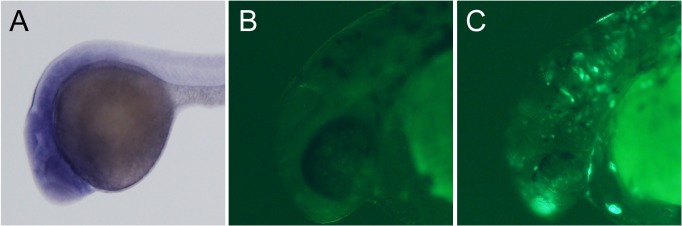
Visualization of endogenous gene expression by knock-in of the eGFP reporter using the crRNA-tracrRNA-Cas9 protein complex. (A) Whole-mount *in situ* hybridization with an *epdr1* probe. (B) An uninjected embryo. (C) The embryo injected two crRNAs (epdr1-crRNA; 25 pg + Mbait-crRNA; 25 pg), tracrRNA (100 pg) and Mbait-hs-eGFP (25 pg) with Cas9 protein (400 pg). The expression of eGFP was detected in the anterior central nervous system, including neurons.

As an application of this system, we investigated whether the expression of an uncharacterized gene could be visualized by the locus-specific knock-in of a reporter construct using the crRNA-tracrRNA-Cas9 protein complex. We recently developed a CRISPR/Cas9-mediated knock-in of a reporter via a homology-independent DNA repair system [21]. Thus, the concurrent cleavage of a donor vector containing the Mbait sequence (targeting by Mbait-gRNA) and the targeted genomic site occurred, and the linearized donor vector was integrated into the target site through a non-homologous end joining pathway. We designed epdr1-crRNA for targeting the initiation codon of an uncharacterized gene *ependymin related 1* (*epdr1*). Two crRNAs (epdr1-crRNA and Mbait-crRNA), tracrRNA and Mbait-hs-eGFP (reporter construct containing the Mbait site, the *hsp70* promoter and eGFP) were injected with the Cas9 protein into zebrafish embryos. Whole-mount *in situ* hybridization with the *epdr1* probe revealed broad *epdr1* expression in the anterior central nervous system (Fig 8A). The eGFP expression in the embryos, in which eGFP integrated into the *epdr1* locus as confirmed by sequencing and genomic PCR using the locus-specific primers (S7 Fig), was detected in neural cells (Fig 8). Thus, the crRNAs-tracrRNA-Cas9 protein complex is suitable for the CRISPR/Cas9-mediated knock-in of a reporter construct into a targeted genomic locus.

## Discussion

The recent evidence suggests that the injection of gRNA and Cas9 mRNA into fertilized embryos efficiently induces DSBs at the targeted genomic locus in various model organisms [31,32]. Recently, Lee J-S et al. reported the genome modification using the purified Cas9 protein and *in vitro* transcribed tracrRNA and gene-specific crRNA [33]. In this study, we presented a ready-to-use CRISPR system that uses synthetic crRNA and tracrRNA together with the recombinant Cas9 protein.

We chose *tyrosinase* and *spns2* loci to assess the genome editing activity of tracrRNA and crRNAs because we already had previously evaluated gRNAs (tyr-gRNA and spns2-gRNA1) [19]. Furthermore, we had not observed any detectable off-target effects of the gRNAs/Cas9 on their potential off-target sites. When two crRNAs (tyr-crRNA and spns2-crRNA1) and tracrRNA with the Cas9 protein were injected into zebrafish embryos, the two expected phenotypes such as cardia bifida and an eye pigment defect were observed with high efficiency (Table 1). These phenotypes were identical to the phenotypes observed with the gRNAs/Cas9 mRNA injections. We confirmed that multiple genome modifications induced by the two crRNAs (tyr-crRNA and spns2-crRNA1) and tracrRNA with the Cas9 protein were heritable (Fig 6). Furthermore, we found that the crRNAs-tracrRNA-Cas9 protein injection induced genome modifications earlier than the crRNAs-tracrRNA-Cas9 mRNA injection (Fig 4). We speculate that recombinant Cas9 protein is more effective than Cas9 mRNA because of its immediate enzymatic activity without any translational delay. Western blotting using anti-Cas9 antibody revealed that Cas9 protein was detected at 1hpf and disappeared at 24hpf in the crRNA-tracrRNA-Cas9 protein system, while the translated Cas9 protein from Cas9 mRNA was marginal at 1 hpf and significantly accumulated at 24 hpf in the crRNA-tracrRNA-Cas9 protein system (Fig 5). The rapid degradation of Cas9 protein in the crRNA-tracrRNA-Cas9 protein system may be an advantageous point to avoid considerable side effects of Cas9 nuclease activity. In fact, we occasionally observed slight morphological defects besides cardia bifida in the embryos injected with the crRNAs-tracrRNA-Cas9 mRNA (S1 Fig).

We noticed that the crRNAs and gRNAs containing identical target sequences do not always demonstrate similar genome editing activity. We found that each crRNAs (spns2-crRNA2, s1pr2-crRNA1 and s1pr2-crRNA2) possessed target sequences that were identical to their respective gRNAs (spns2-gRNA2, s1pr2-gRNA1 and s1pr2-gRNA2) but demonstrated distinct genome editing activity (Fig 7). Thus, the crRNA/Cas9 protein injections triggered mutations in their targets that were more effective than the gRNA/Cas9 protein injections, suggesting that the three-dimensional structure and/or stability of the crRNAs-tracrRNA-Cas9 or gRNA-Cas9 complex may be dependent on the individual target sequence. One possible explanation is that certain target sequences might self anneal during *in vitro* transcription of gRNA from the gRNA expression vectors. A recent crystal structure analysis of Cas9 and the gRNA/Cas9/DNA complex revealed that Cas9 undergoes a dramatic conformational change during the process of Cas9-gRNA-DNA formation [34,35,36]. We propose that the crRNAs-tracrRNA-Cas9 protein complex is a ready-to-use system that can be used as an alternative genome editing tool.

Recently, Auer et al. reported the CRISPR/Cas9-mediated knock-in of a donor vector into the targeted genomic locus by a homology-independent DNA repair [20]. We further improved this method by using a donor vector that contained a bait sequence (Mbait) upstream of the hsp70 promoter-eGFP gene for gRNA-mediated cleavage [21]. It is possible that cis-regulatory elements of the target gene affect the hsp70 promoter when the donor vector is integrated into the upstream region of the target gene, monitoring of the endogenous expression by GFP. Using the crRNA-tracrRNA-Cas9 protein complex, we successfully visualized the cells expressing the uncharacterized *epdr1* gene with eGFP expression under the endogenous enhancer activity in F0 embryos (Fig 8), thereby demonstrating a useful application of this method. We previously reported the establishment of knock-in line by the gRNA-Cas9 mRNA system [21]. The germline transmission of the knock-in allele in the crRNA-tracrRNA-Cas9 protein system should be evaluated in further analysis of the F0 founders.

In summary, we developed a simple, ready-to-use genetic method that consists of synthetic multiple crRNAs, tracrRNA and recombinant Cas9 protein. This simple strategy may become a new standard for introducing genome modifications in zebrafish and may be widely applicable in other model organisms.

## Supporting Information

S1 Fig Phenotypic analysis of the embryos injected with two crRNAs, tracrRNA and Cas9 protein. The injection conditions used in S1 Fig were the same as those used in Fig 1. (A, F, K, P) uninjected embryos. (B, G, L, Q) Tg(*cmlc2*:*EGFP*)-derived embryos injected with spns2-crRNA1, tyr-crRNA, tracrRNA and Cas9 protein. (C, H, M, R) Tg(*cmlc2*:*EGFP*)-derived embryos injected with spns2-gRNA1, tyr-gRNA and Cas9 protein. (D, I, N, S) Tg(*cmlc2*:*EGFP*)-derived embryos injected with spns2-crRNA1, tyr-crRNA, tracrRNA and Cas9 mRNA. (E, J, O, T) Tg(*cmlc2*:*EGFP*)-derived embryos injected with spns2-gRNA1, tyr-gRNA and Cas9 mRNA. The phenotypic results between the samples in Fig 1 and S1 Fig are essentially similar. White and black arrowheads indicate the position of the developing heart and the position of the eye, respectively. The embryos in (A), (B), (C), (D), (E), (K), (L), (M), (N) and (O) correspond to the embryos in (F), (G), (H), (I), (J), (P), (Q), (R), (S) and (T), respectively. (A-E, K-O) Ventral view with anterior at the top at 1 dpf. (F-J, P-T) Lateral view with anterior to the left and dorsal at the top at 2 dpf.
(TIFF)

S2 Fig Requirement of tracrRNA and crRNA for Cas9-mediated genome modifications. (A, E) an uninjected embryo. (B, F) Tg(*cmlc2*:*EGFP*)-derived embryos injected with Cas9 protein (400 pg). (C, G) Tg(*cmlc2*:*EGFP*)-derived embryos injected with Cas9 mRNA (250 pg). (D, H) Tg(*cmlc2*:*EGFP*)-derived embryos injected with two crRNAs (spns2-crRNA; 25 pg + tyr-crRNA; 25 pg) and tracrRNA (100 pg). No abnormality in cardiac development at 1 dpf or the retinal epithelium pigmentation at 2 dpf was observed.
(TIFF)

S3 Fig Sequence analysis of the genome modifications induced by two crRNAs, tracrRNA and Cas9 protein. PCR amplicons for the *spns2*- and *tyr*-target sites from the individual genomic DNA (as shown in S1 Fig) were inserted into the pGEM-Easy vector, and the inserted fragments derived from the individual PCR amplicons were randomly sequenced. The targeted genomic sequences and PAM sequences are indicated by the green and blue letters, respectively. The deleted and inserted nucleotides compared with the wild-type sequence (top row) are indicated by the red dashes and red letters, respectively. The numbers of nucleotides deleted (-) and inserted (+) are indicated to the right with the detection number. Slashes mean a gap in the genome sequence containing a large insertion or a large deletion.
(TIFF)

S4 Fig Sequence analysis of the genome modifications induced by two crRNAs, tracrRNA and Cas9 mRNA. PCR amplicons for the *spns2*- and *tyr*-target sites from the individual genomic DNA (as shown in S1 Fig) were inserted into the pGEM-Easy vector, and the inserted fragments derived from the individual PCR amplicons were randomly sequenced. The targeted genomic sequences and PAM sequences are indicated by the green and blue letters, respectively. The deleted and inserted nucleotides compared with the wild-type sequence (top row) are indicated by the red dashes and red letters, respectively. The numbers of nucleotides deleted (-) and inserted (+) are indicated to the right with the detection number. Slashes mean a gap in the genome sequence containing a large insertion or a large deletion.
(TIFF)

S5 Fig Time course analysis of the genome modifications induced by two crRNAs, tracrRNA and Cas9 protein. PCR amplicons for the *spns2*- and *tyr*-target sites from the individual genomic DNA (as shown in Fig 4) were inserted into the pGEM-Easy vector, and the inserted fragments derived from the individual PCR amplicons were randomly sequenced. The targeted genomic sequences and PAM sequences are indicated by the green and blue letters, respectively. The deleted and inserted nucleotides compared with the wild-type sequence (top row) are indicated by the red dashes and red letters, respectively. The numbers of nucleotides deleted (-) and inserted (+) are indicated to the right with the detection number. Slashes mean a gap in the genome sequence containing a large insertion or a large deletion.
(TIFF)

S6 Fig Phenotypic analysis of the embryos injected with three crRNAs, tracrRNA and Cas9 protein. (A) An uninjected embryo. (B) Tg(*cmlc2*:*EGFP*)-derived embryos injected with tracrRNA (100 pg), spns2-crRNA2 (25 pg), s1pr2-crRNA1 (25 pg) s1pr2-crRNA2 (25 pg) and Cas9 protein (400 pg). (C) Tg(*cmlc2*:*EGFP*)-derived embryos injected with spns2-gRNA1 (25 pg), s1pr2-gRNA1 (25 pg), s1pr2-gRNA2 (25 pg) and Cas9 protein (400 pg). Cardia bifida was observed in the embryo injected with multiple crRNAs, tracrRNA and Cas9 protein, whereas a normal single heart was observed in an uninjected embryo and the embryo injected with multiple gRNAs and Cas9 protein. (A-C) Ventral view with anterior at the top. White arrowheads indicate the position of the developing heart.
(TIF)

S7 Fig Locus-specific integration of the reporter using the tracrRNA-crRNA-Cas9 protein complex. Two crRNAs (epdr1-crRNA; 25 pg + Mbait-crRNA; 25 pg), tracrRNA (100 pg) and Mbait-hs-eGFP (25 pg) were co-injected with Cas9 protein (400 pg) into zebrafish embryos. Genomic DNA was prepared from an uninjected embryo (Fig 6B) and the injected embryo (Fig 6C). (A) Integration of the reporter into the *epdr1* locus was evaluated by genomic PCR. The sizes of the DNA ladder markers are indicated by the base pairs at the left. (B) Sequence of the junction between *epdr1* and the reporter. Nucleotide sequences of the *epdr1* genome, small insertion and the reporter are indicated by the black, red and blue letters, respectively.
(TIF)

S1 Table Sequences of target sites, oligonucleotides and synthetic RNA. (TIFF)

S2 Table PCR Primers used in this study. (TIF)
