# Supplementary figures and images for: Efficient Multiple Genome Modifications Induced by the crRNAs, tracrRNA and Cas9 Protein Complex in Zebrafish
Source: PLoS One. 2015 May 26;10(5):e0128319. doi: 10.1371/journal.pone.0128319 (PMC4444095; doi:10.1371/journal.pone.0128319)

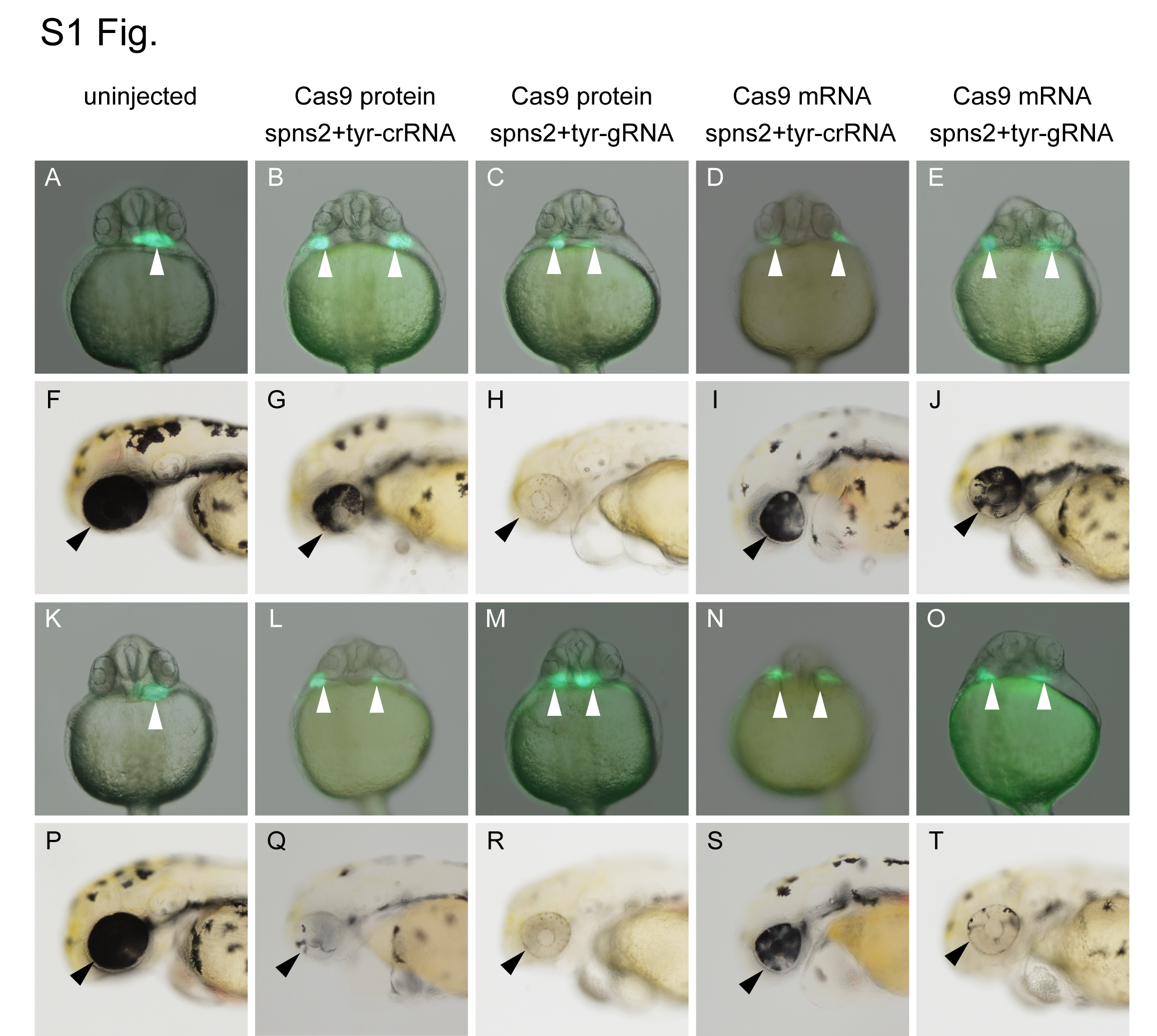

Supplement: S1 Fig — The injection conditions used in S1 Fig were the same as those used in Fig 1. (A, F, K, P) uninjected embryos. (B, G, L, Q) Tg(cmlc2:EGFP)-derived embryos injected with spns2-crRNA1, tyr-crRNA, tracrRNA and Cas9 protein. (C, H, M, R) Tg(cmlc2:EGFP)-derived embryos injected with spns2-gRNA1, tyr-gRNA and Cas9 protein. (D, I, N, S) Tg(cmlc2:EGFP)-derived embryos injected with spns2-crRNA1, tyr-crRNA, tracrRNA and Cas9 mRNA. (E, J, O, T) Tg(cmlc2:EGFP)-derived embryos injected with spns2-gRNA1, tyr-gRNA and Cas9 mRNA. The phenotypic results between the samples in Fig 1 and S1 Fig are essentially similar. White and black arrowheads indicate the position of the developing heart and the position of the eye, respectively. The embryos in (A), (B), (C), (D), (E), (K), (L), (M), (N) and (O) correspond to the embryos in (F), (G), (H), (I), (J), (P), (Q), (R), (S) and (T), respectively. (A-E, K-O) Ventral view with anterior at the top at 1 dpf. (F-J, P-T) Lateral view with anterior to the left and dorsal at the top at 2 dpf. (TIFF) [file pone.0128319.s001.tiff]

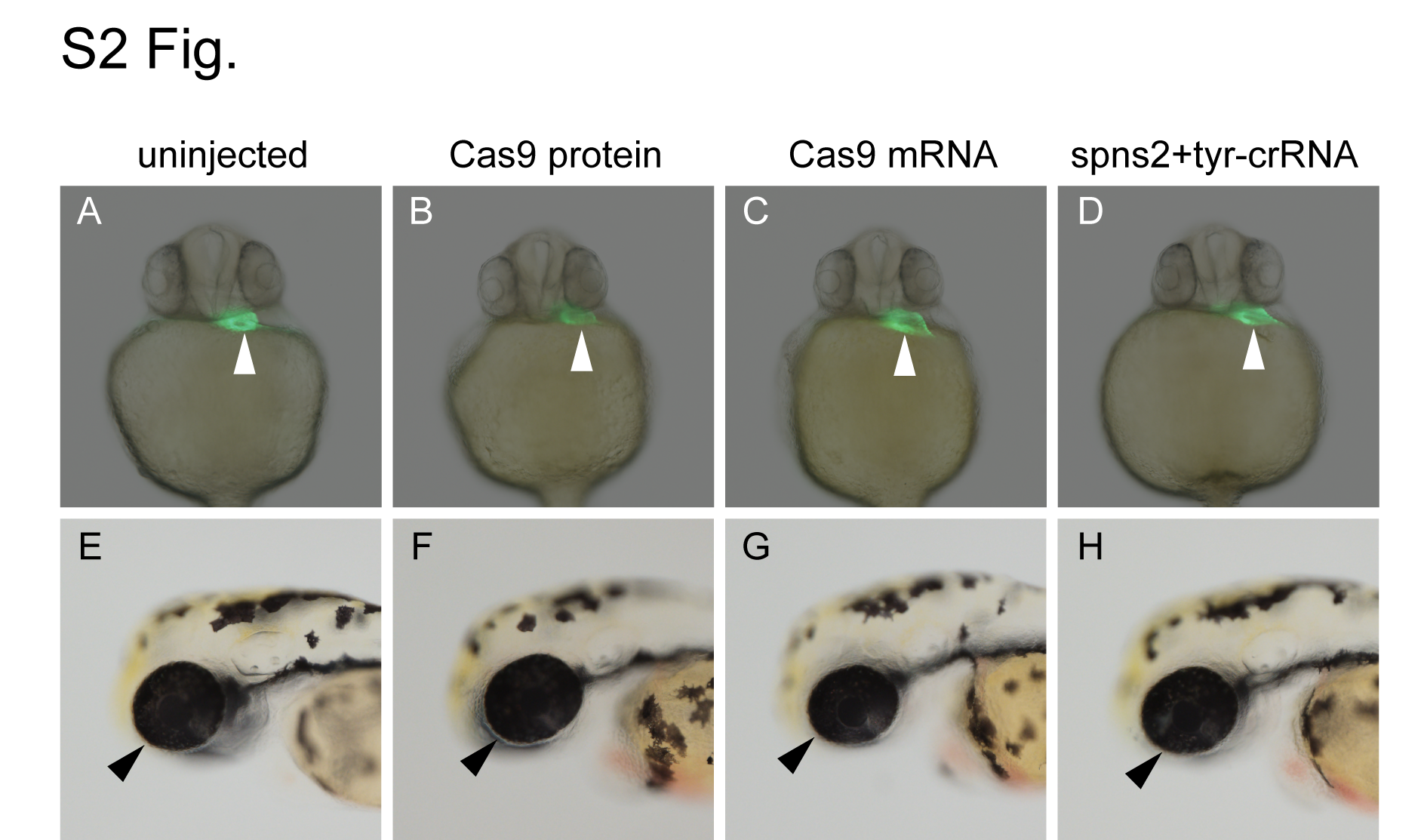

Supplement: S2 Fig — (A, E) an uninjected embryo. (B, F) Tg(cmlc2:EGFP)-derived embryos injected with Cas9 protein (400 pg). (C, G) Tg(cmlc2:EGFP)-derived embryos injected with Cas9 mRNA (250 pg). (D, H) Tg(cmlc2:EGFP)-derived embryos injected with two crRNAs (spns2-crRNA; 25 pg + tyr-crRNA; 25 pg) and tracrRNA (100 pg). No abnormality in cardiac development at 1 dpf or the retinal epithelium pigmentation at 2 dpf was observed. (TIFF) [file pone.0128319.s002.tiff]

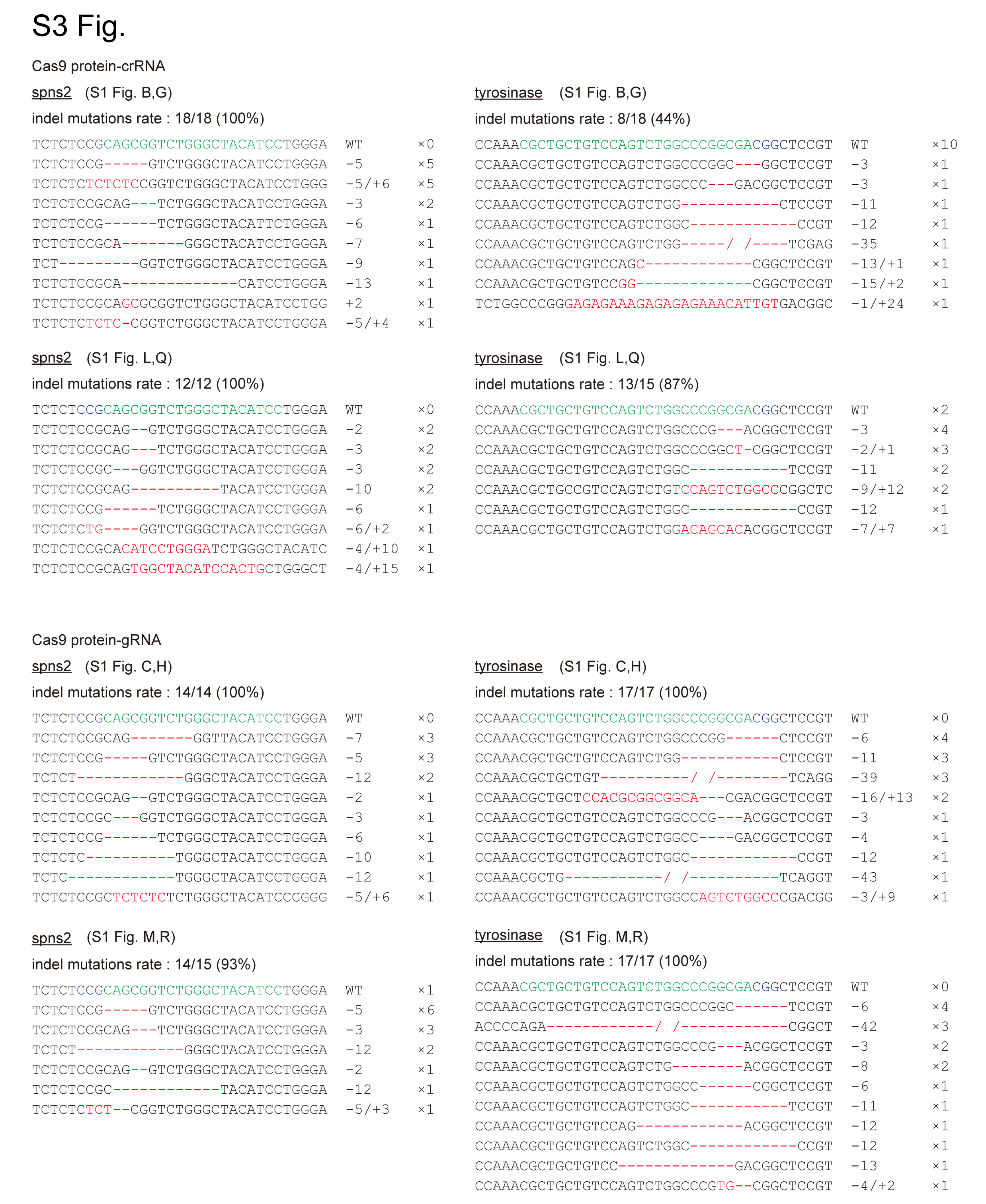

Supplement: S3 Fig — PCR amplicons for the spns2- and tyr-target sites from the individual genomic DNA (as shown in S1 Fig) were inserted into the pGEM-Easy vector, and the inserted fragments derived from the individual PCR amplicons were randomly sequenced. The targeted genomic sequences and PAM sequences are indicated by the green and blue letters, respectively. The deleted and inserted nucleotides compared with the wild-type sequence (top row) are indicated by the red dashes and red letters, respectively. The numbers of nucleotides deleted (-) and inserted (+) are indicated to the right with the detection number. Slashes mean a gap in the genome sequence containing a large insertion or a large deletion. (TIFF) [file pone.0128319.s003.tiff]

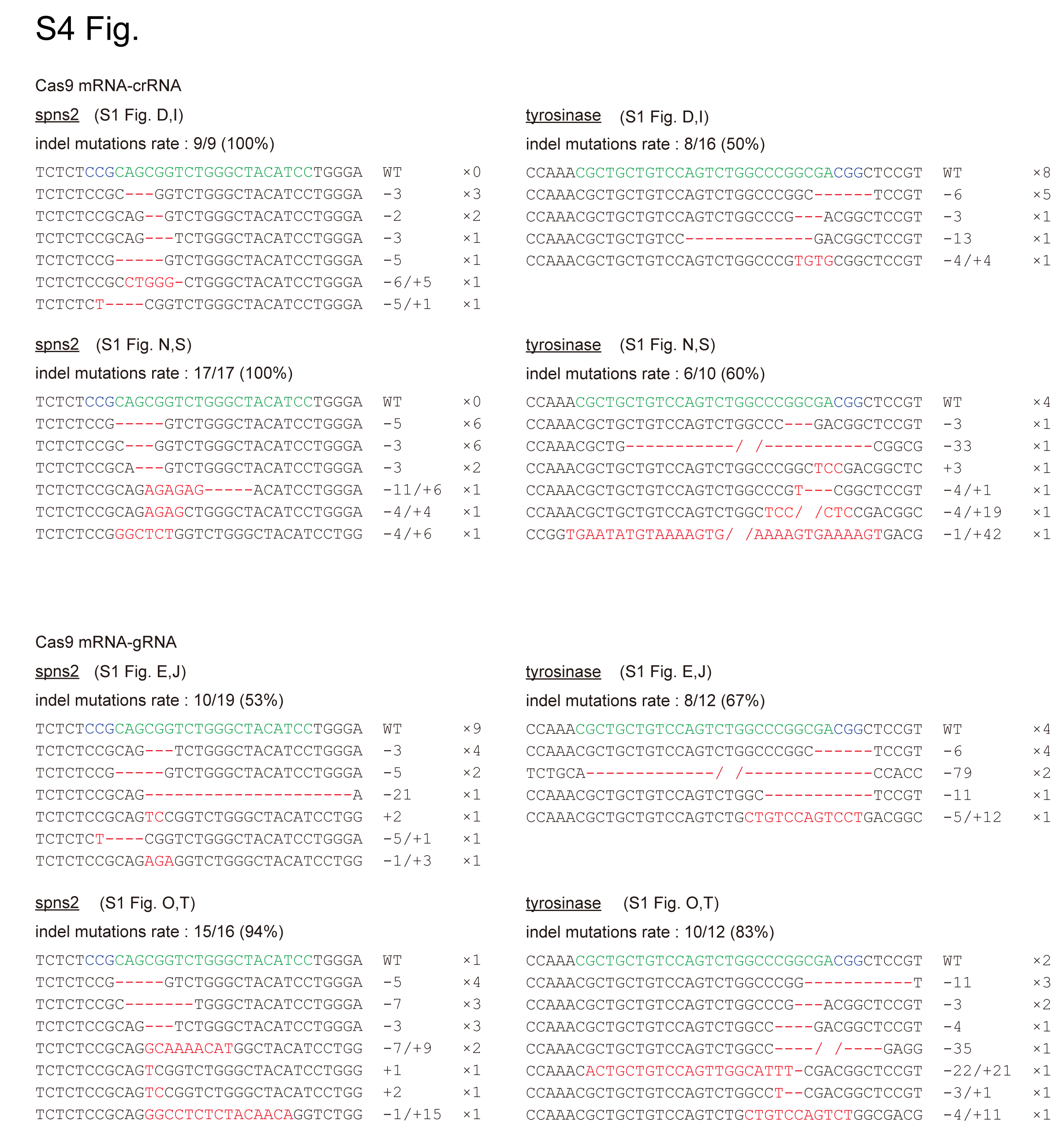

Supplement: S4 Fig — PCR amplicons for the spns2- and tyr-target sites from the individual genomic DNA (as shown in S1 Fig) were inserted into the pGEM-Easy vector, and the inserted fragments derived from the individual PCR amplicons were randomly sequenced. The targeted genomic sequences and PAM sequences are indicated by the green and blue letters, respectively. The deleted and inserted nucleotides compared with the wild-type sequence (top row) are indicated by the red dashes and red letters, respectively. The numbers of nucleotides deleted (-) and inserted (+) are indicated to the right with the detection number. Slashes mean a gap in the genome sequence containing a large insertion or a large deletion. (TIFF) [file pone.0128319.s004.tiff]

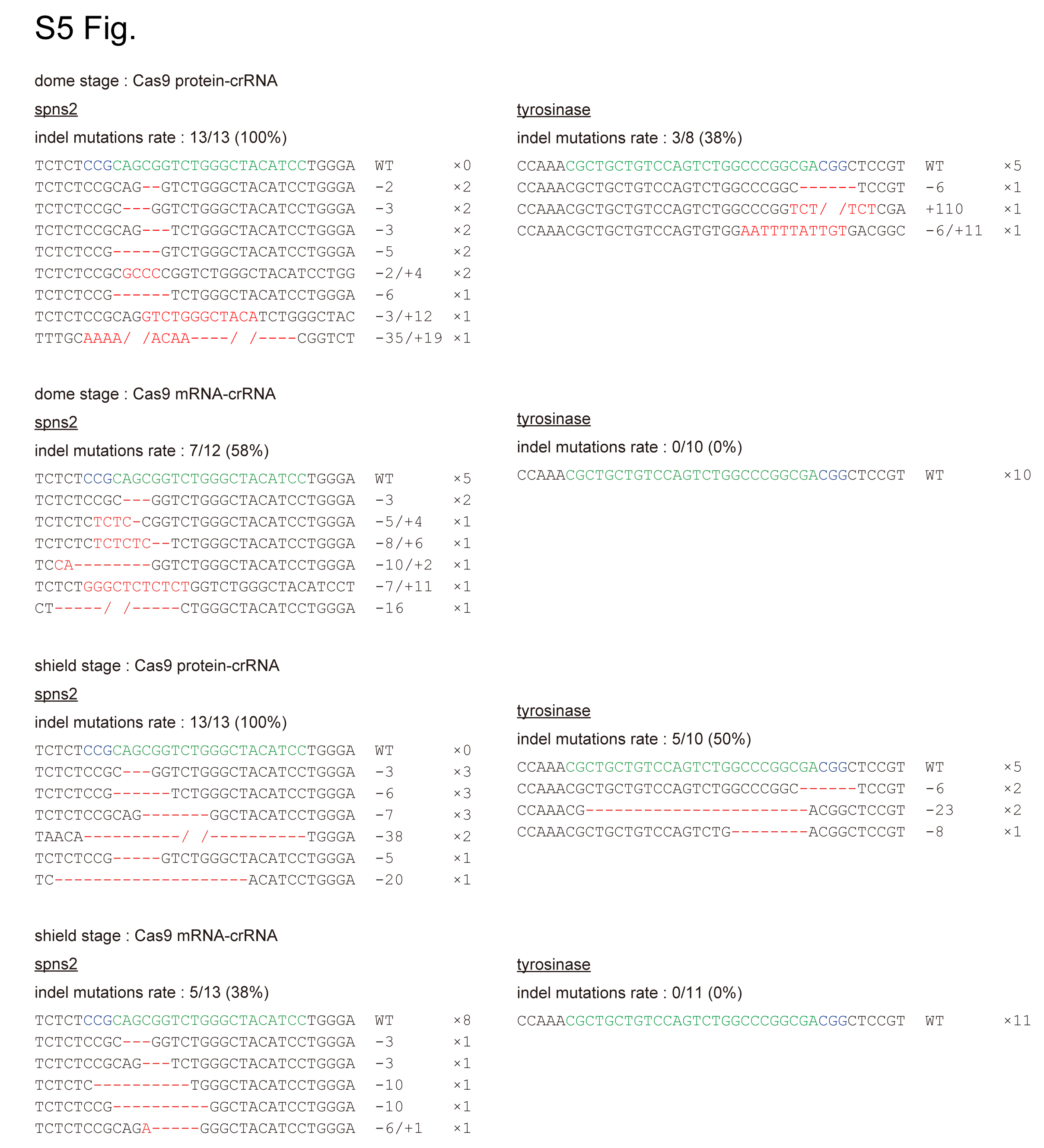

Supplement: S5 Fig — PCR amplicons for the spns2- and tyr-target sites from the individual genomic DNA (as shown in Fig 4) were inserted into the pGEM-Easy vector, and the inserted fragments derived from the individual PCR amplicons were randomly sequenced. The targeted genomic sequences and PAM sequences are indicated by the green and blue letters, respectively. The deleted and inserted nucleotides compared with the wild-type sequence (top row) are indicated by the red dashes and red letters, respectively. The numbers of nucleotides deleted (-) and inserted (+) are indicated to the right with the detection number. Slashes mean a gap in the genome sequence containing a large insertion or a large deletion. (TIFF) [file pone.0128319.s005.tiff]

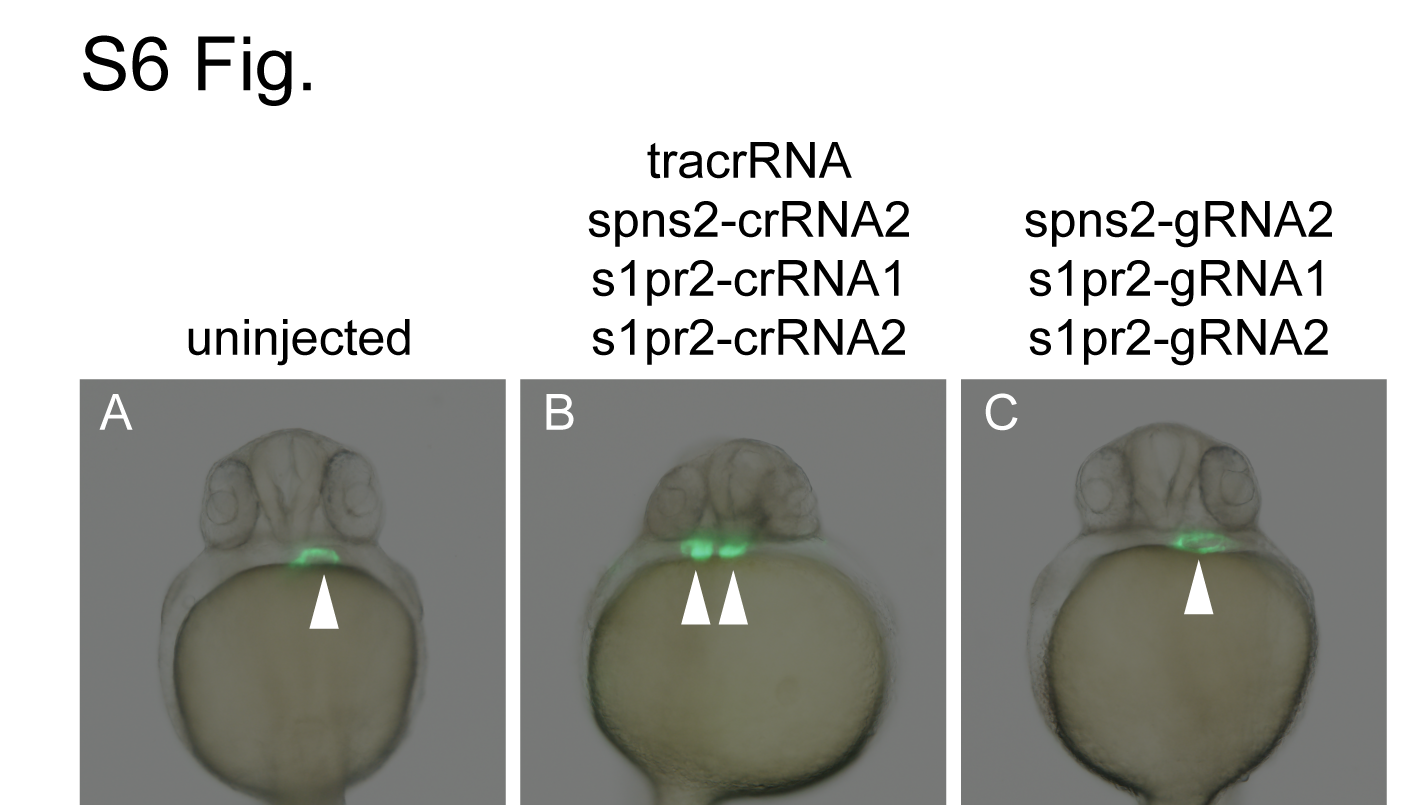

Supplement: S6 Fig — (A) An uninjected embryo. (B) Tg(cmlc2:EGFP)-derived embryos injected with tracrRNA (100 pg), spns2-crRNA2 (25 pg), s1pr2-crRNA1 (25 pg) s1pr2-crRNA2 (25 pg) and Cas9 protein (400 pg). (C) Tg(cmlc2:EGFP)-derived embryos injected with spns2-gRNA1 (25 pg), s1pr2-gRNA1 (25 pg), s1pr2-gRNA2 (25 pg) and Cas9 protein (400 pg). Cardia bifida was observed in the embryo injected with multiple crRNAs, tracrRNA and Cas9 protein, whereas a normal single heart was observed in an uninjected embryo and the embryo injected with multiple gRNAs and Cas9 protein. (A-C) Ventral view with anterior at the top. White arrowheads indicate the position of the developing heart. (TIF) [file pone.0128319.s006.tif]

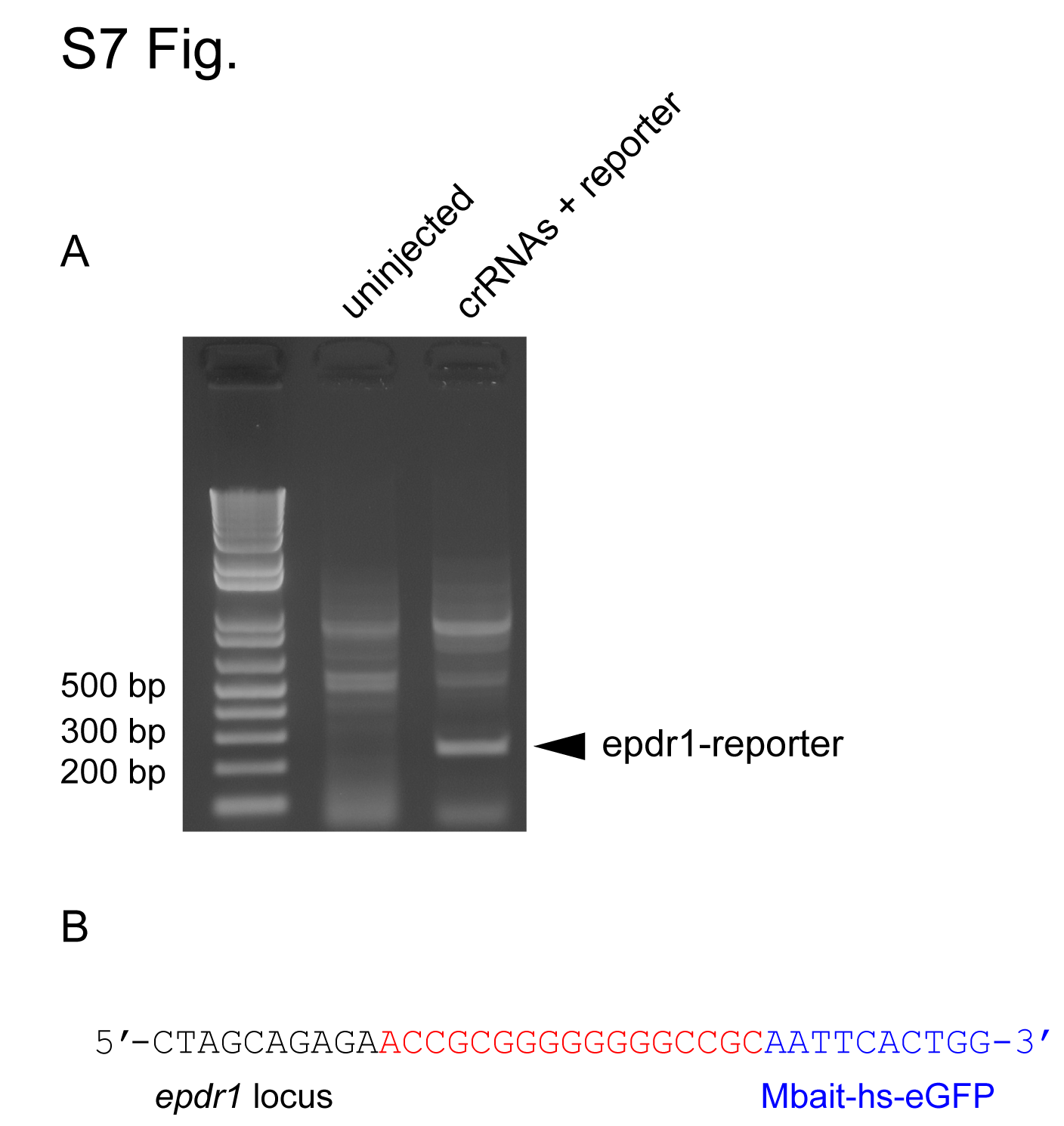

Supplement: S7 Fig — Two crRNAs (epdr1-crRNA; 25 pg + Mbait-crRNA; 25 pg), tracrRNA (100 pg) and Mbait-hs-eGFP (25 pg) were co-injected with Cas9 protein (400 pg) into zebrafish embryos. Genomic DNA was prepared from an uninjected embryo (Fig 6B) and the injected embryo (Fig 6C). (A) Integration of the reporter into the epdr1 locus was evaluated by genomic PCR. The sizes of the DNA ladder markers are indicated by the base pairs at the left. (B) Sequence of the junction between epdr1 and the reporter. Nucleotide sequences of the epdr1 genome, small insertion and the reporter are indicated by the black, red and blue letters, respectively. (TIF) [file pone.0128319.s007.tif]

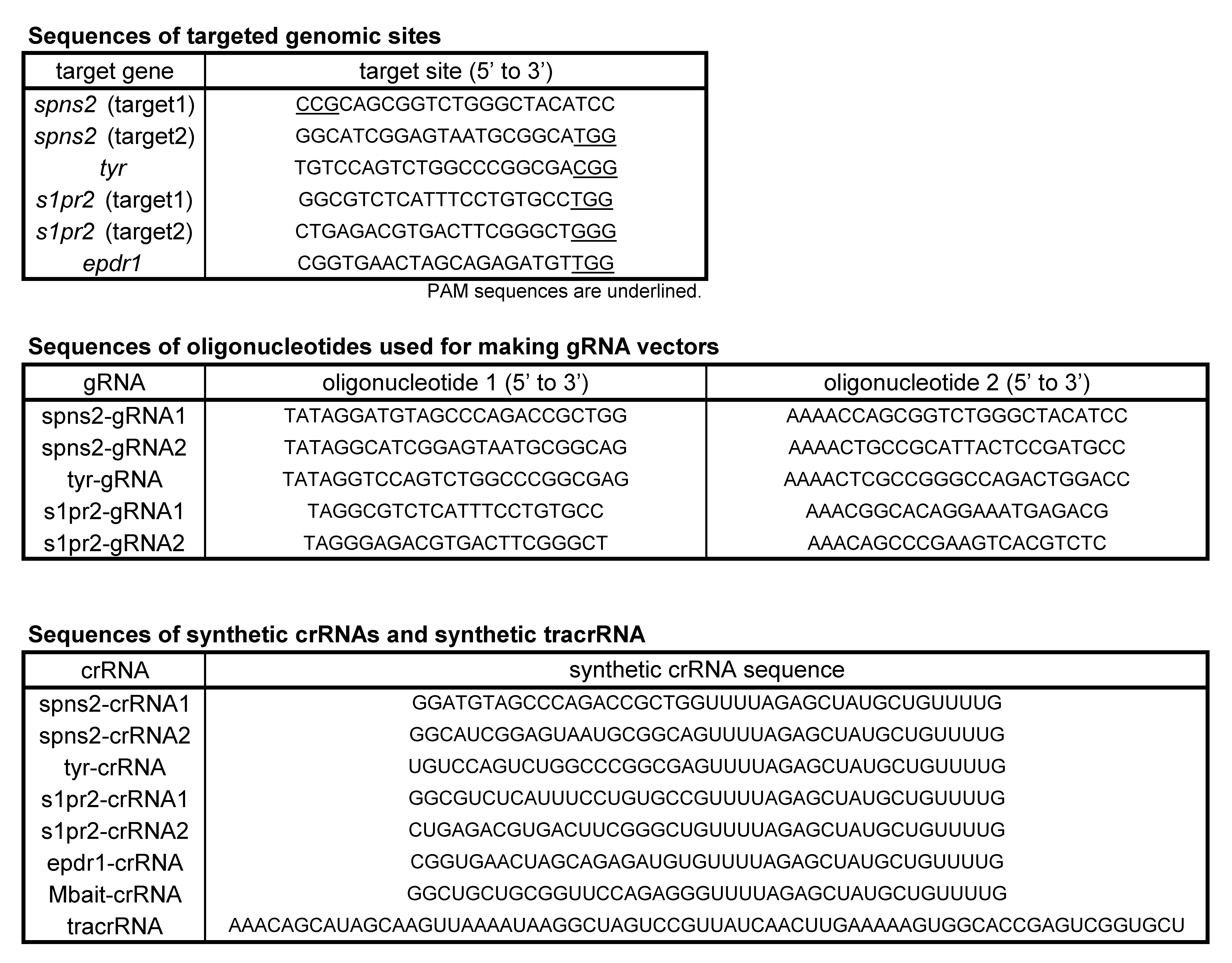

Supplement: S1 Table — (TIFF) [file pone.0128319.s008.tiff]

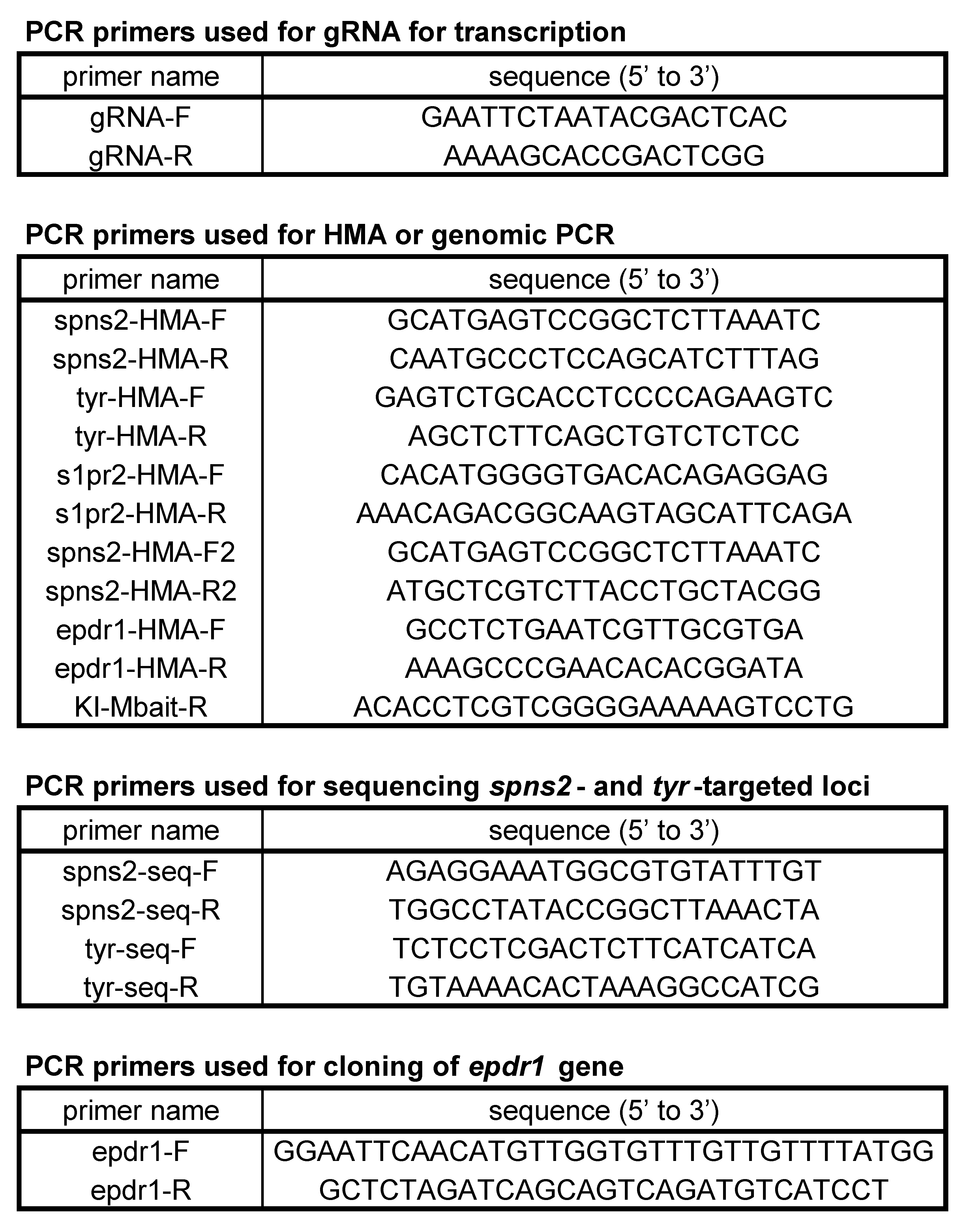

Supplement: S2 Table — (TIF) [file pone.0128319.s009.tif]
